# POMC neurons in heat: A link between warm temperatures and appetite suppression

**DOI:** 10.1371/journal.pbio.2006188

**Published:** 2018-05-07

**Authors:** Maria A. Vicent, Conor L. Mook, Matthew E. Carter

**Affiliations:** Department of Biology, Program in Neuroscience, Williams College, Williamstown, Massachusetts, United States of America

## Abstract

When core body temperature increases, appetite and food consumption decline. A higher core body temperature can occur during exercise, during exposure to warm environmental temperatures, or during a fever, yet the mechanisms that link relatively warm temperatures to appetite suppression are unknown. A recent study in *PLOS Biology* demonstrates that neurons in the mouse hypothalamus that express pro-opiomelanocortin (POMC), a neural population well known to suppress food intake, also express a temperature-sensitive ion channel, transient receptor potential vanilloid 1 (TRPV1). Slight increases in body temperature cause a TRPV1-dependent increase in activity in POMC neurons, which suppresses feeding in mice. Taken together, this study suggests a novel mechanism linking body temperature and food-seeking behavior.

High body temperatures decrease food intake in a variety of mammals, including humans [1–3]. Although normally regulated within a narrow homeostatic range, core body temperature can increase during exposure to high environmental temperatures and following high-intensity exercise. Body temperature also rises during a fever, a pathological condition that can decrease appetite for several days. Although great progress has been made in the past two decades to elucidate the neural bases of thermosensation [4] and appetite [5,6], the biological mechanisms by which warmer body temperatures suppress food intake are unknown.

Multiple organ systems are involved in appetite regulation; however, the motivation to eat is ultimately controlled by the brain [5,6]. Several distinct populations of neurons in the brain measure hormones and nutritional factors in the blood, neural signals from the digestive system, and sensory signals from the environment, including visual and olfactory cues, to maintain energy homeostasis and an ideal body weight. Perhaps the brain region most studied in the central regulation of food intake is the arcuate nucleus of the hypothalamus, which resides adjacent to the third ventricle (Fig 1) [5]. The arcuate nucleus contains two populations of neurons that oppositely regulate appetite: a population that expresses agouti-related peptide (AgRP)—a secreted neuropeptide that stimulates food intake [7]—and a population that expresses pro-opiomelanocortin (POMC)—a protein that is cleaved into multiple secreted neuropeptides, including α-melanocyte stimulating hormone (αMSH), which suppresses food intake [8].

**Fig 1:**
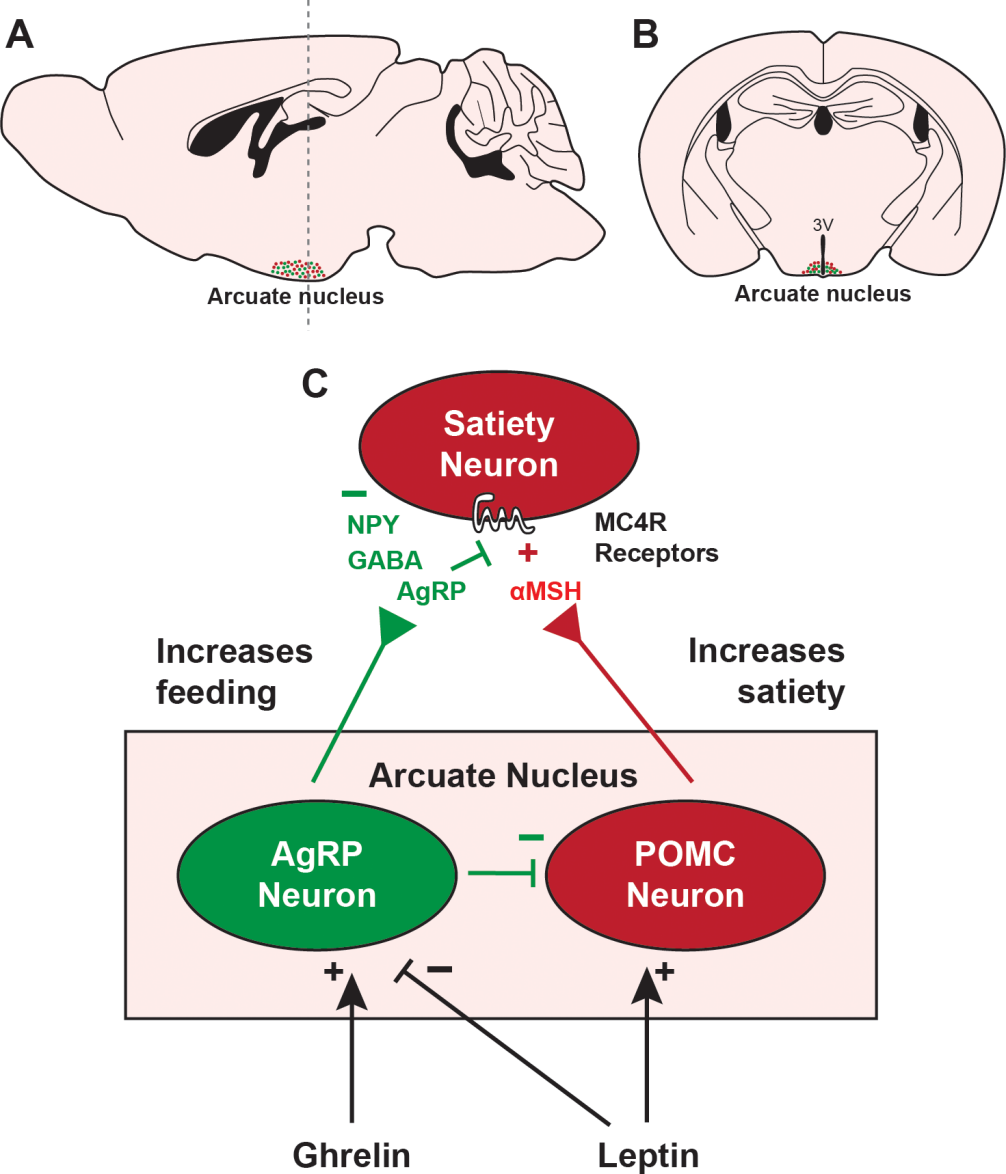
Control of food intake by the arcuate nucleus of the hypothalamus. (A) Sagittal diagram of a rodent brain showing AgRP neurons (green) and POMC neurons (red) in the arcuate nucleus. Dashed vertical line shows coronal plane depicted in B. (B) Coronal diagram of a rodent brain showing AgRP neurons and POMC neurons in the arcuate nucleus adjacent to the 3V. (C) Functional signaling of the arcuate nucleus. The satiety hormone leptin stimulates POMC neurons and inhibits AgRP neurons within the arcuate nucleus. AgRP neurons are also directly stimulated by the orexigenic hormone ghrelin. POMC neurons stimulate downstream satiety neurons by releasing αMSH onto MC4R. AgRP neurons release AgRP, which antagonizes melanocortin receptors. AgRP neurons also release NPY and GABA, which inhibit downstream satiety neurons. 3V, third ventricle; αMSH, α-melanocyte stimulating hormone; AgRP, agouti-related peptide; GABA, γ-aminobutyric acid; MC4R, melanocortin 4 receptor; NPY, neuropeptide Y; POMC, pro-opiomelanocortin.

Many findings over the past two decades have begun to reveal the mechanisms by which AgRP neurons and POMC neurons regulate food intake [5] (Fig 1). POMC neurons are stimulated by appetite-suppressing hormones, such as leptin [9–11], released from adipocytes in proportion to fat stores [12], as well as by visual and olfactory cues about food availability [13,14]. AgRP neurons are inhibited by leptin, although the role of leptin-mediated inhibition of AgRP neurons is controversial and may not actually influence feeding [11]. AgRP neurons are also stimulated by the orexigenic hormone ghrelin [15] as well as visual and olfactory food cues [13,14,16]. When stimulated, POMC neurons release αMSH, which decreases food intake by binding to brain melanocortin receptors, especially melanocortin 4 receptor (MC4R), on downstream neurons that induce satiety [17,18]. When artificially stimulated using optogenetics or chemogenetics, POMC neurons decrease food intake, and these effects depend on melanocortin signaling [19,20]. Interestingly, AgRP blocks MC4R, antagonizing the anorexigenic effects of αMSH [21]. AgRP neurons also corelease neuropeptide Y (NPY) and γ-aminobutyric acid (GABA), which inhibit downstream satiety neurons to increase food intake (Fig 1) [9,22].

The ability of POMC neurons to suppress feeding in response to various internal and external signals makes them good candidates to mediate appetite suppression in response to elevated core body temperature. How might POMC neurons detect an increase in local temperature, and how might they react upon thermal stimulation? In a recent study in *PLOS Biology* [23], Jeong and colleagues test the hypothesis that POMC neuronal activity increases in response to warm temperatures via expression of a thermosensitive cation channel, transient receptor potential vanilloid 1 (TRPV1) [24]. This channel transduces information about heat in the peripheral nervous system (Box 1). TRPV1 channels are also activated by capsaicin, the chemical compound produced by hot peppers that causes the perception of heat [24]. However, the hypothesis that TRPV1 can depolarize POMC neurons is controversial. Previous studies showed little to no expression of TRPV1 receptors in the arcuate nucleus [25,26]. Additionally, TRPV1 receptors have been shown to transduce temperatures at and above 42°C [24], much too high a body temperature for mammals, for which 37°C is normal and 38–39°C is elevated.

**Box 1: Molecular mechanisms of thermosensation in mammals**

How do animals transduce temperature? Much research in the past two decades has focused on the transient receptor potential (TRP) family of ion channels as being the primary transducers of temperature in mammals [4]. These TRP channels are expressed in the peripheral nervous system, in the dorsal root ganglia and cells of the spinal cord, and in various locations throughout the brain.

TRPV1 and TRPV2 ion channels transduce noxious hot temperatures, with TRPV1 channels sensitive to temperatures above 42°C [24] and TRPV2 above 52°C [27]. TRPV1 channels are also sensitive to capsaicin, the chemical compound derived from hot peppers that produces perceptions of heat [24]. TRPV3 and TRPV4 channels transduce warm temperatures in a nonnoxious range, 32–39°C [28] and 27–35°C [29], respectively. Additionally, transient receptor potential subfamily M (TRPM) channels—including TRPM2, TRPM4, and TRPM5—may play a role in transduction of warm temperatures [4]. Nonnoxious cool temperatures from 25–28°C are transduced by the TRPM8 channel [30]. This channel is also activated by menthol, a compound naturally produced by mint leaves that produces perceptions of mild cold [30].

While thermosensitive TRP channels are well known to transduce temperature in the peripheral nervous system and skin, the role of TRP channels in the brain is more uncertain [4,31]. TRP channels may transduce thermal stimuli, yet they may also transduce pressure, osmotic, and inflammatory stimuli. Although discrete populations of neurons, such as those in the median preoptic area of the hypothalamus, are known to detect core body temperature and affect homeostatic thermoregulatory physiology, the transduction mechanism of these neurons is unknown [4,31].

The first achievement of Jeong and colleagues was to show that a subpopulation of POMC neurons indeed express TRPV1 [23]. *Trpv1* mRNA was detected in about two-thirds of POMC neurons; TRPV1 protein was highly localized to POMC neurons in the hypothalamus, with very little TRPV1 expressed in neighboring regions. In electrical recordings from brain slices, capsaicin depolarized and triggered action potentials in POMC neurons, an effect that was blocked by a TRPV1 receptor antagonist. POMC neurons were also depolarized by resiniferatoxin (RTX), another selective TRPV1 agonist. Importantly, raising the temperature of the brain slice to 38°C was also sufficient to induce activity in POMC neurons. These effects were attenuated by pharmacological compounds that block synaptic signaling, demonstrating that capsaicin, RTX, and temperatures above the normal physiological range depolarize POMC neurons directly. Taken together, these experiments provide strong evidence that POMC neurons express functional TRPV1 receptors.

TRPV1-mediated depolarization of POMC neurons occurred at 38°C, a lower temperature than the 42-°C previously reported threshold for TRPV1 activation [24]. Interestingly, Jeong and colleagues also found expression of *Trpv3* and *Trpv4* in POMC neurons, cation channels that open in response to lower temperatures of 32–39°C and 27–35°C, respectively (Box 1) [28,29]. Previous studies indicate that TRPV1 can form functional heteromeric channels with other TRP subunits [32,33]. For example, TRPV1/TRPV3 heteromeric channels have an activation threshold of 34°C [33]. Therefore, it is possible that TRPV1 forms heteromeric channels with TRPV3 and/or TRPV4 in POMC neurons to transduce temperatures at 38°C, within a physiological range. Indeed, when Jeong and colleagues genetically knocked down expression of *Trpv3* and *Trpv4*, POMC neurons were less sensitive to increased temperatures. Therefore, TRPV1 and TRPV3/TRPV4 might form functional “TRPV1-like” receptors capable of depolarizing POMC neurons at temperatures outside the range of TRPV1 channels alone. Future research is necessary to characterize these heteromeric channels at a molecular level and to fully demonstrate an interaction between these different subunits in POMC neurons.

The key question is whether or not these temperature-sensitive POMC neurons cause a decrease in appetite. Indeed, microinjection of capsaicin into the arcuate nucleus caused a decrease in food intake. These effects are specific to TRPV1, as genetic knockdown of *Trpv1* mRNA blocked capsaicin-induced suppression of feeding. These effects are also specific to POMC neurons, as chemogenetic inhibition of POMC neurons or pharmacological blockade of αMSH receptors also blocked capsaicin-induced suppression of feeding. Therefore, TRPV1 expression in POMC neurons is sufficient to mediate capsaicin-induced decreases in food intake.

Finally, Jeong and colleagues asked whether POMC-mediated suppression of feeding occurred during physical exercise, a normal physiological state that raises both core body and brain temperature [34]. Mice were placed on a treadmill for 40 min to mimic a physiological state similar to cardiovascular exercise in humans. Indeed, this exercise raised temperature in the arcuate nucleus to 38–39°C and reduced subsequent feeding for the next hour compared with nonexercised mice. Importantly, the effects of exercise on appetite suppression were attenuated when the *Trpv1* gene was deleted in POMC neurons or when αMSH receptors were blocked. Therefore, functional TRPV1 channels and POMC neuron signaling is required for exercise-induced suppression of food intake.

Taken together, the results of Jeong and colleagues demonstrate that POMC neurons express functional TRPV1-like receptors, that these receptors increase POMC neuronal activity when temperature increases, and that functional activation of POMC neurons is necessary for appetite suppression in response to exercise-induced elevation of core body temperature (Fig 2) [23]. These studies advance our understanding of appetite regulation by providing a link between a thermosensitive ion channel and a specific population of appetite-suppressing neurons. It will be fascinating for future experiments to determine whether POMC neurons are also necessary for the appetite-suppressing effects of increases in body temperature due to environmental temperature and fever. Consistent with these hypotheses, genetic knockout of the *Trpv1* gene attenuates fever responses in mice [35]. TRPV1-mediated activation of POMC neurons would confer high adaptive value during a fever, in which it would be beneficial to rest for faster and efficient healing rather than foraging for food. Additionally, core body temperature fluctuates according to an animal’s circadian rhythm, and it would be interesting to know if POMC-mediated suppression of appetite is influenced by core body temperature at various points during an animal’s active/inactive period. Consistently, *Pomc*^*−/−*^ mice show greatly reduced locomotor activity, an activity essential for foraging, during the active period [36].

**Fig 2:**
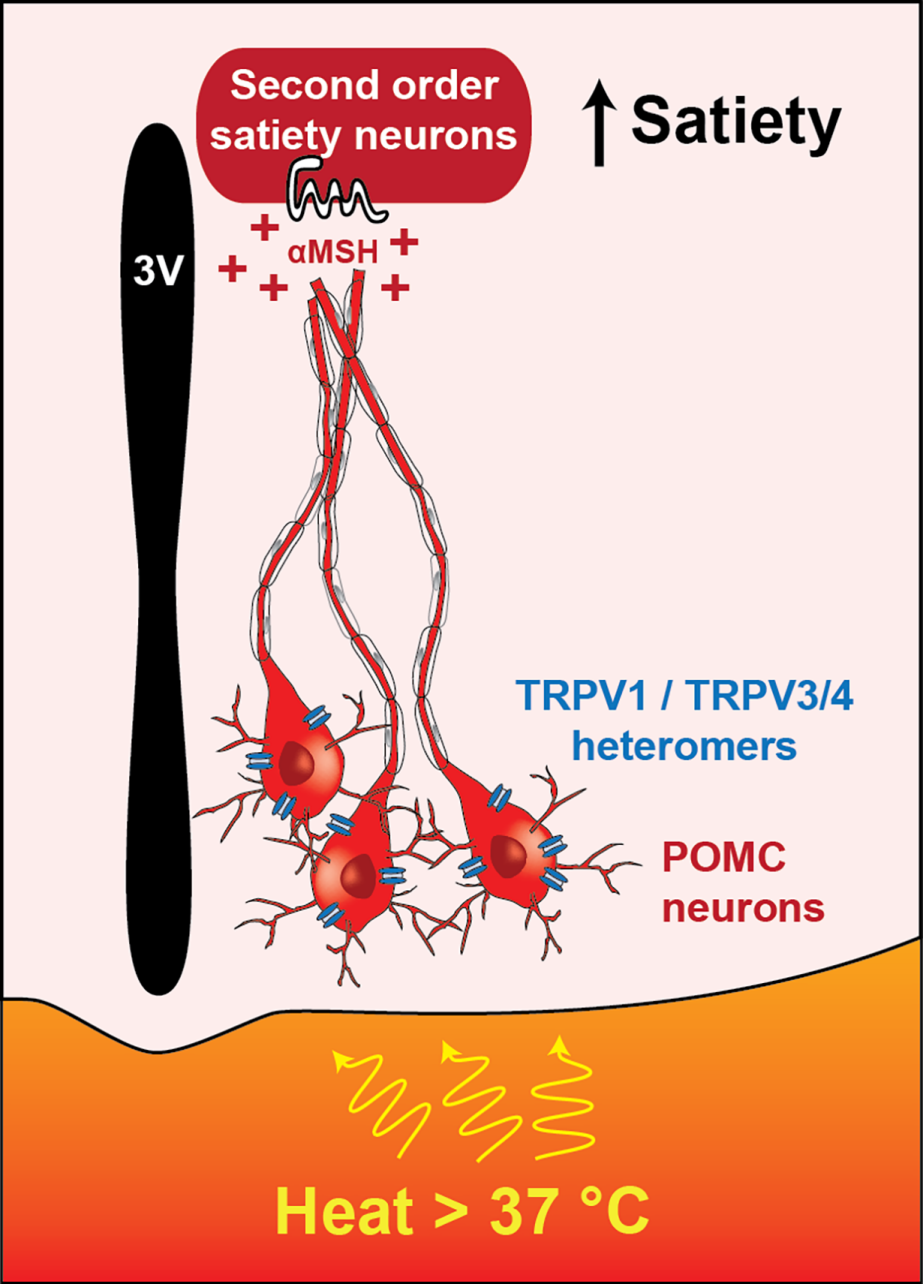
Model for the role of TRPV1-like receptors on POMC neurons in appetite suppression. Elevated core body temperature above 37°C is transduced by POMC neurons via TRPV1-like receptors, heteromers of TRPV1 and TRPV3/4. Increased activity in POMC neurons causes increased melanocortin signaling in downstream second order neurons that promote satiety. 3V, third ventricle; αMSH, α-melanocyte stimulating hormone; POMC, pro-opiomelanocortin; TRPV1, transient receptor potential vanilloid 1.

Interestingly, the results of Jeong and colleagues are consistent with previous studies demonstrating that POMC neurons mediate other causes of appetite suppression. For example, nicotine administration decreases appetite and facilitates weight loss in humans and a variety of model organisms [37,38]. Nicotine increases neural activity in POMC neurons via activation of α3β4 nicotinic acetylcholine receptors, which consequently decrease food intake in mice [39]. Genetic suppression of α3β4 receptors exclusively on POMC neurons blocks nicotine’s effects on appetite suppression in a strikingly similar way to how genetic suppression of *Trpv1* exclusively in POMC neurons blocks the effects of increasing hypothalamic temperature on appetite suppression [39]. These complementary results demonstrate that POMC neurons can be directly stimulated not only by endogenous satiety hormones but also by nicotine and warm temperatures in a physiological range, allowing for nonendocrine factors to reduce appetite.

Evolutionarily, a heat detector in anorexigenic POMC neurons would also be of high adaptive value during competing need states. For example, body temperature acutely increases in high-intensity situations such as aggression, escape behavior, and sexual encounters. In each of these behavioral states, hunger and food intake must be adaptively postponed to ensure survival of the organism. Therefore, TRPV1-mediated increases in POMC neuron activation would suppress food-seeking behavior to maximally promote survival.

Previous studies that stimulated arcuate POMC neurons demonstrated that several hours of stimulation is necessary to reduce food intake [19,20]. These effects are in contrast with the results of Jeong and colleagues, in which stimulation of TRPV1-expressing POMC neurons decreased food intake within minutes. To explain this discrepancy, Jeong and colleagues suggest heterogeneity among the POMC neuron population throughout the arcuate nucleus. Indeed, individual POMC neurons have been shown to be anatomically and functionally distinct [40]. Perhaps TRPV1-expressing POMC neurons are functionally efficient in reducing food intake, while others paradoxically promote feeding. For example, a distinct population of arcuate POMC neurons was recently shown to express the cannabinoid type 1 receptor (CB1R), and CB1 agonists depolarized POMC neurons and caused an increase in feeding [41]. Therefore, it is plausible that TRPV1- and CB1R-expressing POMC neurons represent functionally antagonistic subpopulations; stimulating only the TRPV1-expressing population might suppress food intake more quickly and efficiently than stimulating the entire heterogeneous group.

Another interesting question for future investigation is the degree to which temperature-sensitive ion channels regulate neurons that orchestrate other behaviors, including increases in appetite, thirst, sexual behavior, and aggressive behavior. For example, appetite is not only suppressed by warm environmental temperatures but also elevated by cold environmental temperatures [2,3]. Perhaps other thermosensitive ion channels mediate these effects on AgRP neurons or other neurons that regulate energy homeostasis. The present study demonstrates the feasibility and plausibility of these experiments, suggesting some hot topics for future research.

## Abbreviations

αMSH: α-melanocyte stimulating hormone
AgRP: agouti-related peptide
CB1: cannabnoid type 1
CB1R: cannabinoid type 1 receptor
GABA: γ-aminobutyric acid
MC4R: melanocortin 4 receptor
NPY: neuropeptide Y
POMC: pro-opiomelanocortin
RTX: resiniferatoxin
TRP: transient receptor potential
TRPM: transient receptor potential subfamily M
TRPV: transient receptor potential vanilloid
